# A German Taptoo

**Published:** 1888-11-10

**Authors:** 


### A German Taptoo.

What, in the first place, is a German Taptoo? Who makes it, how is it made, and is it for eating, or drinking, or only for looking at? Only for looking at, it must be admitted, but it is a thing well worth seeing, a spectacle that seems almost too bright or vivacious for our stolid islanders, and would better befit a more southern clime. But Tommy Atkins—for it is the British soldier who gets up and performs in the German Taptoo—has not worn his red coat, or blue as the case may be, nor gone through his drill without acquiring an appreciation of the value of colour and measured motion, and a taptoo which was recently got up in honour of the Duke of Cambridge by the Royal Marine Artillery at the Eastney Barracks, Portsmouth, deserves to be recorded, not only because it was so successful, but because it was got up and carried through by the men themselves without aid or advice from their officers. The Royal Marine Artillery is at present under the command of one of the ablest officers in the service, Colonel Crease, C.B., who is not only a fine soldier and smart officer, but an accomplished scientist and inventor, who has conferred great benefits on her Majesty's navy by more than one of his ingenious inventions. And now for the Taptoo!

Three bands stood in the centre of a hollow square formed by 200 men, each bearing a lighted torch. Outside these were 250 soldiers with fixed swords, who were followed by 400 men carrying Chinese lanterns, and 300 without—a gallant corps of 1,250 in all. These marched round the parade-ground the bands playing various popular airs, and on passing the officers' mess, whence the imposing pageant was witnessed by his Royal Highness and the officers of the corps and their friends, all hands cheered loudly. The square then halted in the middle of the parade, where it formed a circle whilst the bands played a descriptive piece entitled, "An Episode in a Soldier's Life,' which gave a musical illustration of the réveillè, the alarm, and the "first shot" leading to the engagement, which was enlivened by volleys of real musketry, fired by men specially told off for the purpose. Meanwhile the lantern-bearers had filed off, and entering the barracks, had put a couple of lanterns outside each window.

When this performance was over, the lanterns were withdrawn, and amid perfect silence a choir of 250 soldiers' children, accompanied by soft music, sang half of the first verse of "God Save the Queen." After them the thousand soldiers took up the strain, and sang it lustily to the accompaniment of all the bands. Then the children sang the second half of the first verse—"Send her victorious"—and again the manly chorus thundered forth the words, in which the spectators, who by this time had assembled to the number of 8,000, joined "with heart and voice," whilst 300 blue lights, one of which was placed in each window in the barracks, gave a weird illumination to a spectacle which those who witnessed it will never forget.

As an effect merely of colour and sound, this German taptoo would be worthy of the study and imitation of that great man whom *Punch* habitually calls Augustus Druriolanus; but to the Duke of Cambridge and the other spectators it was the more interesting and touching in that it was an honest and spontaneous expression of the affectionate esteem in which he is held by the men of the Marine Artillery and the service generally, and of that loyalty to the Throne and Royal Family which—when all that malcontents and agitators can say or do, is said and done—burns deeply and strongly in all British hearts.

